# Editorial

**Published:** 1861-01

**Authors:** 


### Editorial.

### GOLD.

The first article in the present number of the Register, on the treatment of gold, by Prof. Calvert, is good. There are, however, some little points upon which he might have been a little more explicit, or more understandable to the common reader, or those but little acquainted with chemistry.

In the preparation of aqua regia, he says: "Into a florence flask put by weight, nitric acid 38° B 25 parts, and hydrochloric acid 21° B 30 parts." We think the better method for those who are not adepts in chemistry, is the following: Take nitric and hydrochloric acids, both chemically pure, and by measure, one part of the former and four to six parts of the latter. The less nitric acid employed the better, so it accomplishes the work, which is simply by its presence to liberate the chlorine.

After the gold is dissolved, even though it is a saturated solution, there may be free nitric acid which should be driven off by evaporation; indeed, this is necessary for the most perfect condition of the solution.

He says in regard to precipitation, that the precipitant should be added as long as any precipitate continues to fall. Now, we think it quite as correct a test and more convenient, to add the precipitant till effervescence ceases, then the chlorine has formed its new combination, the gold is then necessarily all free and will be precipitated.

He remarks: "Should the iron be added very rapidly, the probability is, that the deposit will be less heavy." According to our experience, the opposite is true, when the precipitant is added most rapidly the precipitate is most heavy.

The remarks upon the treatment of gold with silver, are very good. The entire article should be carefully studied by all.

T.

### FIFTEENTH VOLUME.

This number begins the fifteenth volume of the Dental Register. This journal, like many kindred things, has passed through several changes and vicissitudes, some of which, at least we hope, have been for the better.

The Register started out a quarterly, the volume containing 227 pages, with good and valuable matter for the times; it extended through eight volumes, varying but little from that size. The ninth was enlarged to 448 pages, and was well filled with interesting and valuable matter. It was, each year, a little enlarged, till the thirteenth volume, when it became a monthly, and expanded to 672 pages. Volume fourteen consists of six numbers, making about 400 pages. Volume fifteen will consist of twelve numbers, making about 800 pages.

Now we have no boast to make in regard to what the Register has been, for those who have read it know full as well as we. We have only to say, that so far as our humble ability would admit, we have endeavored to gather up the fragmentary items of professional progress, and put them in such form as will be most tangible. For the future we promise nothing more than is indicated by the past. T.

### DISEASED ANTRUM.

Sept. 29th, 1860, Mr. M.——called for an examination of diseased antrum. He is of good constitution, and of nervous-bilious temperament. His health had been good until within a few weeks, during which time his strength and appetite have been failing. He has suffered to some extent for a year past, in the right antrum of Highmore. There has been almost a constant discharge of pus from the nostril of that side; there has also been frequent discharges of blood in small quantities. The pus discharged was often times of a greenish cast, and very fœtid. The pain has been gradually increasing; there has been, however, no external soreness or tumefaction. The first superior molar of that side, had some four or five decayed cavities in its crown, by none of which, however, was the pulp exposed, and it was still living, and so far as the vital connection of the tooth was concerned, it was perfectly healthy. No soreness was exhibited on percussion. The tooth was, therefore, not the cause of the difficulty in the antrum. But, the tooth being so much decayed, and having no antagonist, removal was decided upon in order to effect an opening in the antrum. After the tooth was removed, the floor of the antrum was perforated by an opening about two lines in diameter. A small quantity of thick pus was discharged through the orifice; washed out the antrum with tepid water having in it a little tincture of myrrh and chloride of soda. The passage from the antrum into the nostril afforded a free passage of the fluid. After thoroughly cleansing out the antrum, proceed with a blunt-pointed probe to make an examination of the cavitiy; nothing unusual was indicated upon the walls, but upon the superior part of the roof of the cavity, there was either a tumor or extensive thickening of the mucous membrane; this was indicated by the yielding of the probe, to the extent of about a line.

During the first two days, washed out the antrum once every six hours with the diluted tincture of myrrh, with a few drops of creosote added.

After syringing out, the opening was closed with a piece of soft wood dressed to the proper size; this was done to prevent the closing of the opening. Each time after removing the plug of wood there was a free discharge of pus; this, however, gradually became less. I directed the patient to use for a wash, the simple tincture of myrrh, with about thirty drops of tincture of iodine to an ounce of myrrh, and if there was much fetor, to use occasionally an injection, chloride of soda, or the tincture of myrrh, with a few drops of creosote, and to take such means as will best establish the general health, using good nourishing food, and taking ample exercise in the open air. T.

### "A. B."

Is our friend, "A. B.," of the American Dental *Review*, surprised that he was expected to be candid and truthful as an editor? If so, we can understand most of his "whew" editorial in the November number of the *Review;* if not, it is quite beyond our capacity. He says: "We are even accused of intentional misrepresentations." Certainly, friend "A. B.," and convicted of it, too, by your persistent neglect to correct your misrepresentation. His palaver about Webster and Worcester is all right, perhaps, but amounts to nothing; and there is no special call in Providence for his hope that he will be able to express his ideas more clearly. No one, that we know of, complains of any want of *clearness* in the expression of his ideas. Want of *fairness* in expressing the ideas of others, is all that the Register has objected to. It would have required less space than is occupied by his late editorial, to make an honorable, straightforward correction of his mistake; (?) but that would have let all his readers see how grossly he had misrepresented the language and sentiments of Prof. Taft, while professing to make a verbatim quotation from his article. W.

"Render unto Cesar."—The American Dental *Review*, by oversight, of course, credits the New York Dental *Journal* with Prof. Calvert's article on "Artistic Dentistry." By a careful reading of the *Journal*, our *Review* brethren will see that it obtained it from the *Register.* W.

### BOUCHET'S PLUGGERS.

We not long since had occasion to speak of and direct the attention of the profession to the Dental instruments manufactured by Bouchet, of New York, especially drills and excavators, and we now, with much satisfaction, refer to his pluggers. We have been using them for several months and find them in every way superior to anything we had used before. The points are of the different sizes and shapes required, and finely and perfectly serrated, and work smoothly in the gold without any hanging or dragging. The steel of which they are manufactured, is either of a very superior quality or wrought by some method to us unknown. We have operated with these instruments till we do not know how we could get along with those in common use, the results obtained with fine instruments are so much more efficient, beautiful and satisfactory, than those obtained by the use of inferior ones, that we can hardly conceive how any one will tolerate imperfect instruments. The best are always cheapest, (cost what they may) and the most satisfactory to all concerned. Much more depends upon good plugging instruments than most dentists are willing to concede. The thorough consolidation of a plug, as well as its thorough adaptation to the walls of a cavity, depends quite as much upon the perfection of the instruments as the skill with which they are used.

T.

---

SALIVARY GLANDS IN THE BREAST.—"The most serious objection that can be advanced to chewing tobacco, is its extreme filthiness, an objection which should be sufficient to prevent its use in that way among dentists. The next is the drain upon the salivary glands, but among old chewers this is so slight as to be of no practical moment; they expectorate but little, and old smokers not at all." *Salivary* glands drained by *expectoration!* But we must not be too critical, for the writer has told us that this was "written with the smoke of a good cigar curling around my head." The mind, like the eye, can not see very distinctly through smoke. For further particulars, see an article in the December number of the *Cosmos*, by Chas. Woodnutt, D. D. S.

W.

### "ARGENTINE"! (?)

"Monsieur Tonson's come again!"

ACCORDING to Garrick, one item in the direction given by Jupiter to his attendants when about to make Goldsmith, was,

"Set fire to the head and set fire to the tail."

Fortunately, the "M." editor of the American *Review* has adopted this principle, in the illumination of an article in his November number. The article is about "ARGENTINE," whatever that is. By the headlight of the article, we are able to read "Gold versus Amalgam," and by its caudal phosphoresence, we are able to discern a very ordinary method of preparing "amalgam paste," from which we infer that "argentine" is the rotten carcass of amalgam cement, long since buried "with the burial of an ass," by the better informed of the profession, but now resurrected and *nom de plumed* by this editor of the *Review*. "Argentine," indeed! We have heard of a kind-hearted man who never calls a certain long-eared animal a mule, lest the name might suggest that he is the son of a jackass. He calls him a *horseine*.

But seriously, we are surprised at M's article, and we are surprised at the man. He gives a case in which two bicuspid teeth were filled—one with gold, the other with amalgam cement. By means of clasps and a plate, the two plugs were electrically connected by a perfect metallic conductor. After a lapse of two years, or more, the tooth filled with gold is found decayed around the plug, while the other remains sound. Now, while this is exactly what science would look for, under the circumstances, and while it affords one of the strongest arguments against the use of amalgams, our friend goes on to dignify them with a high-sounding name, and to defend the use of them, in view of this case. His opinion is that the main reason for the "seeming superiority" of amalgam over gold is, that it "exerts a chemical, as well as mechanical influence to arrest caries." Well, what kind of chemical influence does it exert? Dental caries is produced by acids. Is amalgam cement alkaline? In this paragraph, he claims for the amalgam only a "*seeming* superiority" over gold, and tells us that when properly prepared, it "is, *perhaps*, more certain than gold to stay the ravages of decay" in soft, badly organized teeth. (The italics ours.) He seems, however, to be frightened at such a claim, (and well he might be,) and goes on to state clearly his views, "for fear that some young and inexperienced practitioner may be misled by the foregoing remarks," and conclude that he advocates the indiscriminate use of amalgams. These views are so clearly stated, and are so interesting in themselves, that we will reproduce them. He says, on page 189:—

"In the first place, we admit that many failures have been, and are constantly being made, with amalgam, as a filling for teeth; but we deny, at the same time, that the per centage of failures is greater in this than gold work. And we believe, furthermore, that if the same care in preparing the cavity and the regard to nice manipulation were manifested in filling with argentine that there is with gold, the per centum of success would be larger with the former than the latter. And again, were we to use it in just such cases as we use gold, *i. e.*, in the most favorable cases, the proportion of successful operations would be still greatly augmented. But who that can fill a tooth *well* with gold will resort to anything else, in a case where he feels that he can do such a job as will benefit his patient, and do himself and profession credit?

Could every one who calls himself a dentist do good filling with gold, and were our patients so well aware of the necessity of seeing their dentist early and frequent, then, indeed, would the necessity rarely exist for the employment of anything but pure gold foil. But while the great majority of dentists do not know how, or lack the ability to fill teeth well with gold, and patients neglect their teeth, and refrain from calling on the dentist until their teeth are in an advanced stage of caries, argentine must hold a prominent place in dental therapeutics."

Now, let us look at this. He claims that amalgam filling, as it has been and is practiced, is *as successful* as gold filling. He claims, farther, that if the two styles of work were performed with equal care, amalgam filling would be *more successful* than gold. And, besides, it is used only in unfavorable cases. If used in the favorable cases, as gold is, it would still be *far more successful.* In short, it is as successful as gold, now: If used, with care, it would be more successful. And, if used with care, and in favorable cases, it would be much more so. And this, dear reader, is the doctrine he has stated so "clearly" for fear that some

"young and inexperienced practitioner" will conclude that he advocates "the indiscriminate use of argentine." Now, "young and inexperienced practitioners," don't you use amalgam cement "indiscriminately," or, if you do, be sure to call it "ARGENTINE." To be sure, it saves unfavorable cases, even when carelessly used, just as well as gold, carefully used, saves favorable cases. But don't you use it "indiscriminately."

We stated that we are surprised at M's article, and at the man. At the article for the sentiments it contains, and at the man for professing to hold these sentiments, while he objects to their reduction to practice. What! shall we not use this "argentine," right straight along, when the success of even those "that can fill a tooth *well* with gold" will be augmented? But—pshaw!

W.

THE HIPPOPOTAMUS WITH THE TOOTH ACHE.—We find the following in a London paper:

Mr. Bartlett has kindly sent me the following particulars, which I am sure will be read with great interest. The operation does Mr. Bartlett great credit:

F. T. BUCKLAND—My dear Mr. Buckland, I had intended to write to you before I left town, but could not find time. You will be glad to know that I have succeeded in performing perhaps the largest, if not the greatest dental operation on record. Our male hippopotamus has been, as you know, suffering from a fractured tooth, and fearing that the consequences might be serious, I have had a strong oak fence fixed between his pond and the iron railings, and I then determined to remove the broken tooth; this I accomplished on the morning of Wednesday last, but not without a fearful struggle. I had prepared a powerful pair of forceps, over two feet long; with these I grasped his fractured incisor, thinking, with a firm and determined twist, to gain possession of that fine piece of ivory. This, however, was not so easily done, for the brute, amazed at my impudence, rushed back, tearing the instrument from my hands, and, looking as wild as a hippopotamus can look, charged at me just as I recovered my forceps. I made another attempt, and this time held on long enough to cause the loose tooth to shift its position, but was again obliged to relinquish my hold. I had, however, no occasion to say, "Open your mouth," for this he did to the fullest extent; therefore I had no difficulty in again seizing the coveted morsel, and this time drew it from his monstrous jaws. One of the most remarkable things appeared to me to be the enormous force of the air when blown from the dilated nostrils of this great beast while enraged. It came into my face with a force that almost startled me. A. D. BARTLETT.

---

A FEW days ago we saw the tooth referred to on page 617 of Vol. 13, of the Register, in describing the case of Mrs. C. As some of our readers may not have the volume at hand, we will briefly recapitulate. In May, 1859, Mrs. C. called with an upper incisor badly decayed, pulp dead, and abscess formed. The formation of the abscess had taken place several months previous to her visit. This case was treated, and the teeth filled, at a single sitting. The abscess healed at once, and has never returned, and the gums no longer give evidence that it ever existed. The tooth has given no discomfort since, and its color is better than when first filled. *The canal was not filled*—with gold, or any thing else. This patient has very bad health, but is careful of her teeth. W.

---

*The Pocket Anatomist. Being a Complete Description of the Anatomy of the Human Body, for the use of Students. By* M. W. HILLES.

THIS is a little work upon anatomy, prepared expressly for the student, and is truly *multum in parvo.* It is a valuable work, and one that would be of advantage to all. It is frequently the case with the student that he wishes a leading idea or particular in regard to a part or organ, and a few words will lead to the point he desires, and everything else he desires comes readily to mind.

The work before us is just calculated to accomplish this, and every student of anatomy should have it, or such an one, always at hand.

It is published by Lindsay & Blakiston, of Philadelphia, in a very neat and well finished manner. T.

---

EAST SAGINAW, DEC., 1860.

*Dear Sir :*—The Sixth Annual Meeting of the Michigan Dental Association will be held at Ann Arbor, on Tuesday, the 8th day of January next, at 7 o'clock, P. M.

The interest in these meetings of the Dental Profession is yearly increasing, and no one who wishes to keep posted in the improvements of the Art can fail to be well repaid for attending. Every member of the Association is expected to be present.

Respectfully yours, L. G. WHITING, *Sec'y.*

